# Increased Pre-Transplant Carotid Intima-Media Thickness Is Associated with Early Post-Transplant Atrial Fibrillation, Stroke, and Reduced Survival After Heart Transplantation

**DOI:** 10.3390/life15101539

**Published:** 2025-10-01

**Authors:** Karsten M. Heil, Rasmus Rivinius, Matthias Helmschrott, Ann-Kathrin Rahm, Philipp Ehlermann, Norbert Frey, Fabrice F. Darche

**Affiliations:** Department of Cardiology, Angiology and Pneumology, Heidelberg University Hospital, Im Neuenheimer Feld 410, 69120 Heidelberg, Germany

**Keywords:** atherosclerosis, COPD, graft failure, ischemic cardiomyopathy, mortality, stroke

## Abstract

Background: Carotid intima-media thickness (CIMT) is an established risk factor for adverse cardiovascular events in the general population, but its impact on patients after heart transplantation (HTX) remains unknown. We investigated the effects of an increased pre-transplant CIMT > 0.9 mm on outcomes after HTX. Methods: This observational retrospective single-center study included 311 patients receiving HTX at Heidelberg Heart Center between 2002 and 2014. Patients were stratified by degree of pre-transplant CIMT (CIMT ≤ or >0.9 mm, threshold defined by ESC guidelines). Analysis covered donor and recipient demographics, post-transplant medications, mortality (including causes of death after HTX), early post-transplant atrial fibrillation (AF), and stroke after HTX. Results: A total of 37 of 311 HTX recipients (11.9%) had a pre-transplant CIMT > 0.9 mm. These patients showed an increased 10-year post-transplant mortality (81.1% versus 41.2%, *p* < 0.001) and had a higher percentage of death due to graft failure (24.3% versus 10.6%, *p* = 0.017), as well as due to thromboembolic events/bleeding (10.8% versus 2.9%, *p* = 0.019). Multivariate analysis demonstrated pre-transplant CIMT > 0.9 mm as an independent risk factor for 10-year mortality after HTX (HR: 2.599, 95% CI: 1.683–4.014, *p* < 0.001). Secondary outcomes showed a significantly higher rate of 30-day post-transplant AF (27.0% versus 10.9%, *p* = 0.006) and 30-day stroke after HTX (10.8% versus 1.1%, *p* < 0.001) in patients with a pre-transplant CIMT > 0.9 mm. Conclusion: Pre-transplant CIMT > 0.9 mm is a prognostic marker for early post-transplant AF, stroke, and reduced long-term survival after HTX. Preventive measures, including close monitoring and management of cardiovascular risk factors, are warranted in these high-risk patients.

## 1. Introduction

Heart transplantation (HTX) has been the treatment of choice for patients with irreversible end-stage heart failure for several decades [1,2,3,4,5]. Despite the remarkable advances in surgical techniques, immunosuppressive drug therapy, and post-transplant care, HTX recipients are exposed to multiple long-term cardiovascular challenges after HTX, including cardiac allograft vasculopathy (CAV), graft rejection, atrial fibrillation (AF), and stroke [6,7,8,9,10,11]. However, as CAV, characterized by diffuse atherosclerosis in the graft’s coronary arteries, and other atherosclerosis-associated diseases often remain subclinical for many years, early detection and adequate risk stratification play a key role in post-transplant management [6,7,8,9,10,11].

Carotid intima-media thickness (CIMT), the combined thickness of the intimal and medial layer of the carotid artery wall, is a widely used non-invasive marker of subclinical atherosclerosis which can be measured simply using ultrasound [12,13,14,15,16]. According to the European Society of Cardiology guidelines [17,18,19], a CIMT > 0.9 mm is considered abnormal and is associated with a higher risk of cardiovascular events. As atherosclerosis is a systemic disease, the degree of CIMT can provide insight into the degree of systemic atherosclerosis, reflecting the overall burden of cardiovascular risk factors such as hypertension, dyslipidemia, diabetes, and smoking [12,13,14,15,16,17,18,19]. An increased CIMT is associated with lipid deposition, inflammatory cell infiltration, smooth muscle cell proliferation, arterial stiffening, endothelial dysfunction, and the development of atherosclerotic plaques which can cause plaque rupture, thromboembolism, and vessel occlusion [12,13,14,15,16,17,18,19].

Although studies have investigated the impact of increased CIMT in the general population [20] as well as in specific cohorts such as patients with type 2 diabetes [21] or patients undergoing off-pump coronary artery bypass surgery [22], data regarding the clinical relevance of an increased CIMT in HTX recipients are limited [23,24].

Given the distinct risk profile of HTX candidates, a pre-transplant CIMT > 0.9 mm may serve as a simple and valuable prognostic marker for identifying patients at elevated risk for poor post-transplant outcomes. These patients may benefit from more aggressive preventive strategies, including lifestyle modification and/or pharmacological treatment that could mitigate adverse post-transplant outcomes such as graft failure, cardiovascular events, and reduced survival after HTX. We therefore sought to investigate the effects of an increased pre-transplant CIMT > 0.9 mm on post-transplant outcomes focusing on survival, AF, and stroke after HTX.

## 2. Patients and Methods

### 2.1. Patients

Ethical approval for this study was granted by the institutional review board (IRB) of Heidelberg University, Heidelberg, Germany (ethics approval number: S-286/2015, Version 1.2, 28-07-2020), in accordance with the Declaration of Helsinki. Patients provided written informed consent for their inclusion in the Heidelberg HTX Registry and for the use of their clinical and scientific data. In line with the approved ethics protocol, no additional consent was needed for this observational study since it involved the analysis of only routine clinical data [25,26,27,28,29,30].

We screened the available medical data of all adult patients (≥18 years) who underwent HTX at Heidelberg Heart Center, Heidelberg, Germany, between 2002 and 2014 for pre-transplant carotid intima-media thickness (CIMT) measurements. Patients who had undergone repeat HTX were excluded. Study cohort size was determined by the inclusion period and data availability, rather than by a prespecified power calculation. Measurement of CIMT by carotid ultrasound scan in a supine position was routinely carried out as part of the HTX evaluation and listing process. Assessment of CIMT was performed in accordance with the Mannheim Carotid Intima-Media Thickness Consensus [31]. Patients were stratified based on the results of the carotid ultrasound scan and divided into two groups: patients with a pre-transplant CIMT ≤ 0.9 mm and patients with a pre-transplant CIMT > 0.9 mm. CIMT > 0.9 mm was regarded as abnormal, a predefined cut-off from cardiovascular prevention guidelines, because no HTX-specific cut-off exists [17,18,19].

### 2.2. Follow-Up

Follow-up of HTX recipients was performed in accordance with Heidelberg Heart Center’s routine clinical protocol [25,26,27,28,29,30]. After hospital discharge following HTX, patients were seen monthly as outpatients in the HTX clinic during the first six post-transplant months, then bimonthly until the end of the first year after HTX, and approximately three to four times per year thereafter. From five years after HTX onward, routine follow-up visits were reduced to once or twice annually (with additional visits as clinically needed) [25,26,27,28,29,30].

Routine post-HTX follow-up consisted of several components: medical history, systolic and diastolic blood pressure measurement, a resting 12-lead ECG, and endomyocardial biopsy. Additional assessments included blood and laboratory tests (with immunosuppressive drug monitoring), physical examination, echocardiography, an annual 24 h Holter monitor, and an annual chest X-ray. Complete follow-up data was available for every patient, as there were no losses to follow-up [25,26,27,28,29,30].

### 2.3. Post-Transplant Medication

Administration of post-transplant medication—which includes immunosuppressive pharmacotherapy—took place according to the center standard [25,26,27,28,29,30]. Patients routinely received an initial anti-thymocyte globulin-based immunosuppression induction therapy after HTX as per protocol. Most patients were on an immunosuppressive regimen of tacrolimus and mycophenolic acid. This was because the initial regimen of cyclosporine A and mycophenolic acid was replaced by the tacrolimus and mycophenolic acid combination starting in 2006. All patients also received prednisolone, which was gradually tapered and discontinued six months after HTX if clinically feasible [25,26,27,28,29,30].

### 2.4. Statistical Analysis

Data analysis was performed with MedCalc (Version 23.2.1, MedCalc Software Ltd., Ostend, Belgium). Results are presented as mean ± standard deviation (SD) or as a count (*n*) with a percentage (%). We used mean difference (MD) with 95% confidence interval (CI) for measures of association. Depending on the data and research question, we applied the Student’s *t*-test, Mann–Whitney U-test, analysis of variance (ANOVA), Kruskal–Wallis test, chi-squared test, or Fisher’s exact test. The Kaplan–Meier estimator with a log-rank test was used to graphically compare survival after HTX between patients with a pre-transplant CIMT of ≤0.9 mm and those with a CIMT of >0.9 mm. All visualizations were created using CorelDRAW Graphics Suite 2025 (Version 26.0.0.101; Corel Corporation, Ottawa, ON, Canada). Statistical significance was defined as a *p*-value of <0.050 [25,26,27,28,29,30].

We conducted large-scale univariate analyses to identify differences between patients with a pre-transplant CIMT ≤ 0.9 mm and those with a CIMT > 0.9 mm. The variables analyzed included recipient data, recipient principal diagnosis for HTX, previous open-heart surgery, donor data, transplant sex mismatch, perioperative data, immunosuppressive drug therapy, and post-transplant concomitant medications [25,26,27,28,29,30].

The primary outcome of this study was 10-year mortality after HTX between patients with a pre-transplant CIMT ≤ 0.9 mm and patients with a pre-transplant CIMT > 0.9 mm. Causes of death within ten years after HTX were categorized into the following groups: graft failure, acute rejection, infection/sepsis, malignancy, and thromboembolic event/bleeding. Cause of death was determined from clinical records. Analysis of 10-year post-transplant mortality further included a multivariate analysis (Cox regression model) to investigate the impact of nine variables which were statistically significant in the univariate analysis: recipient age, recipient arterial hypertension, recipient dyslipidemia, recipient diabetes mellitus, recipient chronic obstructive pulmonary disease (COPD), recipient history of smoking, recipient coronary artery bypass graft (CABG) surgery before HTX, recipient ischemic cardiomyopathy (CMP) as principal diagnosis for HTX, and recipient pre-transplant CIMT > 9 mm. We did not include additional variables, such as donor data, in this multivariate analysis for 10-year mortality after HTX to avoid biased regression coefficients and to ensure a stable number of events (deceased patients) per analyzed variable [25,26,27,28,29,30].

Secondary outcomes included analysis of 30-day atrial fibrillation after HTX, 30-day rejection episode after HTX, 30-day TIA after HTX, and 30-day stroke after HTX between patients with a pre-transplant CIMT ≤ 0.9 mm and patients with a pre-transplant CIMT > 0.9 mm. To test the robustness of our findings and investigate a potential era effect related to the change in immunosuppressive regimens from 2006, we performed a sensitivity analysis. This analysis was performed on a subgroup of patients receiving tacrolimus and mycophenolic acid [25,26,27,28,29,30].

## 3. Results

### 3.1. Demographic and Clinical Characteristics

We included a total of 311 HTX recipients in this study after applying the exclusion criteria. A total of 274 of 311 HTX recipients (88.1%) had a pre-transplant CIMT ≤ 0.9 mm while 37 of 311 HTX recipients (11.9%) had a pre-transplant CIMT > 0.9 mm.

Patients with a pre-transplant CIMT > 0.9 mm had a higher recipient age (56.5 ± 6.2 years versus 51.3 ± 10.9 years, *p* < 0.001), a higher percentage of arterial hypertension (75.7% versus 53.3%, *p* = 0.010), a higher percentage of dyslipidemia (78.4% versus 61.7%, *p* = 0.047), a higher percentage of diabetes mellitus (51.4% versus 30.7%, *p* = 0.012), a higher percentage of COPD (54.1% versus 22.6%, *p* < 0.001), and a higher percentage of history of smoking (75.7% versus 54.7%, *p* = 0.016) compared to patients with a pre-transplant CIMT ≤ 0.9 mm. Additionally, patients with a pre-transplant CIMT > 0.9 mm more often received CABG surgery before HTX (32.4% versus 10.6%, *p* < 0.001) and more frequently suffered from ischemic CMP as principal diagnosis for HTX (64.9% versus 30.6%, *p* < 0.001) in comparison to patients with a pre-transplant CIMT ≤ 0.9 mm. There were no statistically significant differences between both groups with respect to donor data, transplant sex mismatch, or perioperative data (all *p* ≥ 0.050). Demographic and clinical characteristics are given in Table 1.

### 3.2. Initial Post-Transplant Medications

Analysis of the immunosuppressive drug therapy showed no statistically significant differences between patients with a pre-transplant CIMT ≤ 0.9 mm and patients with a pre-transplant CIMT > 0.9 mm regarding the use of cyclosporine A, tacrolimus, everolimus, azathioprine, mycophenolic acid, or steroids (all *p* ≥ 0.050). Likewise, we observed no statistically significant differences between both groups regarding the administration of acetylsalicylic acid, angiotensin-converting-enzyme inhibitors/angiotensin II receptor blockers, beta blockers, calcium channel blockers, diuretics, ivabradine, statins, or gastric protection drugs (all *p* ≥ 0.050). Initial post-transplant medications are shown in Table 2.

### 3.3. Post-Transplant Primary Outcome

In terms of the primary outcome of this study, patients with a pre-transplant CIMT > 0.9 mm showed a significantly higher 30-day mortality after HTX (18.9% versus 3.6%, *p* < 0.001), 1-year mortality after HTX (59.5% versus 17.5%, *p* < 0.001), 2-year mortality after HTX (70.3% versus 20.4%, *p* < 0.001), 5-year mortality after HTX (72.9% versus 28.8%, *p* < 0.001), and 10-year mortality after HTX (81.1% versus 41.2%, *p* < 0.001). Details about the post-transplant primary outcome are provided in Table 3.

**Figure 1:**
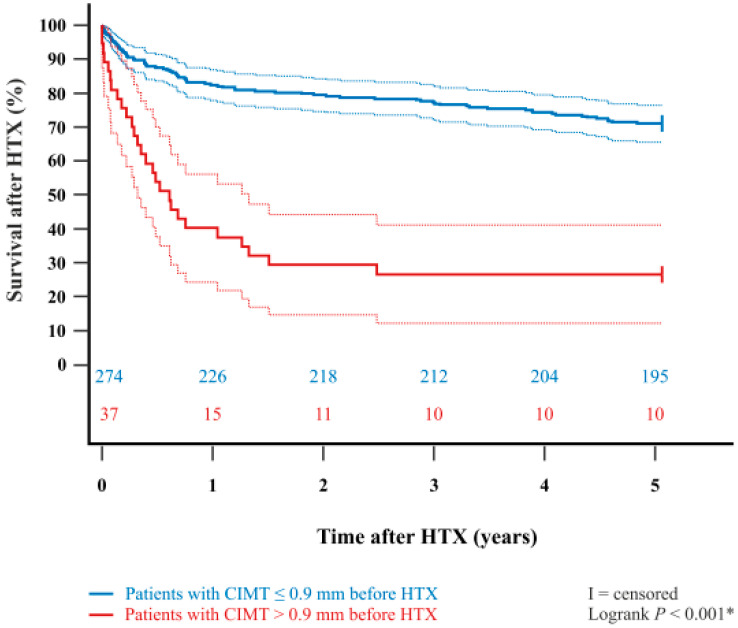
Five-year survival after HTX between patients with CIMT ≤ 0.9 mm or CIMT > 0.9 mm before HTX (Kaplan–Meier estimator). Patients with a pre-transplant CIMT > 0.9 mm had a significantly lower 5-year survival after HTX than patients with a pre-transplant CIMT ≤ 0.9 mm (*p* < 0.001). Dashed lines represent the 95% confidence interval around the respective survival curve. Abbreviations: CIMT = carotid intima-media thickness; HTX = heart transplantation; * = statistically significant (*p* < 0.050).

**Figure 2:**
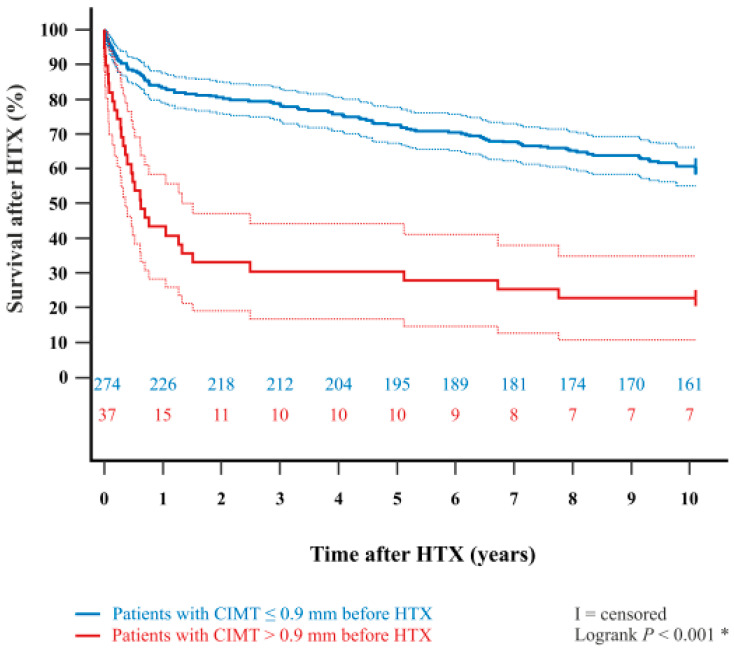
Ten-year survival after HTX between patients with CIMT ≤ 0.9 mm or CIMT > 0.9 mm before HTX (Kaplan–Meier estimator). Patients with a pre-transplant CIMT > 0.9 mm had a significantly lower 10-year survival after HTX than patients with a pre-transplant CIMT ≤ 0.9 mm (*p* < 0.001). Dashed lines represent the 95% confidence interval around the respective survival curve. Abbreviations: CIMT = carotid intima-media thickness; HTX = heart transplantation; * = statistically significant (*p* < 0.050).

In addition, the Kaplan–Meier estimator showed a significantly worse 5-year post-transplant survival (*p* < 0.001) and 10-year post-transplant survival (*p* < 0.001) in patients with a pre-transplant CIMT > 0.9 mm in comparison to patients with a pre-transplant CIMT ≤ 0.9 mm. Kaplan–Meier estimators are displayed in Figure 1 and Figure 2.

When examining causes of death, significantly more patients with a pre-transplant CIMT > 0.9 mm died from graft failure within five years after HTX (21.6% versus 8.0%, *p* = 0.009) and within ten years after HTX (24.3% versus 10.6%, *p* = 0.017) in comparison to patients with a pre-transplant CIMT ≤ 0.9 mm. Patients with a pre-transplant CIMT > 0.9 mm also more frequently died from infection/sepsis within five years after HTX (37.8% versus 16.4%, *p* = 0.002) and within ten years after HTX (37.8% versus 20.4%, *p* = 0.017), as well as from thromboembolic events/bleeding (10.8% versus 1.5%, *p* = 0.001) and within ten years after HTX (10.8% versus 2.9%, *p* = 0.019). No significant differences were observed between the two groups regarding acute rejection or malignancy at 5- or 10-year follow-up after HTX (all *p* ≥ 0.050). Table 4 highlights the causes of death within five and ten years after HTX.

Multivariate analysis for post-transplant mortality showed that pre-transplant CIMT > 0.9 mm was an independent risk factor for a more than twofold increased mortality within five years after HTX (HR: 2.899, 95% CI: 1.802–4.664, *p* < 0.001) and within ten years after HTX (HR: 2.599, 95% CI: 1.683–4.014, *p* < 0.001). In addition, COPD was an independent risk factor for a more than fourfold increased mortality within five years after HTX (HR: 4.748, 95% CI: 2.850–7.910, *p* < 0.001) and within ten years after HTX (HR: 4.695, 95% CI: 3.098–7.115, *p* < 0.001), whereas the other seven included variables (recipient age, recipient arterial hypertension, recipient dyslipidemia, recipient diabetes mellitus, recipient history of smoking, recipient CABG surgery before HTX, and recipient ischemic CMP as principal diagnosis for HTX) showed no statistically significant effect on 5-year or 10-year post-transplant mortality. The multivariate analysis for 5-year and 10-year mortality after HTX is shown in Table 5.

### 3.4. Post-Transplant Secondary Outcomes

In terms of secondary outcomes, patients with a pre-transplant CIMT > 0.9 mm had a significantly higher rate of 30-day atrial fibrillation after HTX (27.0% versus 10.9%, *p* = 0.006) and a significantly higher rate of 30-day stroke after HTX (10.8% versus 1.1%, *p* < 0.001) than patients with a pre-transplant CIMT ≤ 0.9 mm. We observed no significant differences between both groups regarding 30-day TIA after HTX (0.0% versus 0.0%) and 30-day rejection episode after HTX (10.8% versus 13.1%, *p* = 0.691). Details about the post-transplant secondary outcomes are provided in Table 6.

### 3.5. Sensitivity Analysis

To account for the long study period, we performed a sensitivity analysis on a sub-group of patients to check for a possible era effect. This subgroup consisted of the 225 of 311 (72.3%) HTX recipients who received tacrolimus and mycophenolic acid for immunosuppression. The analysis yielded comparable results, supporting the robustness of our findings and suggesting that an era effect was unlikely.

## 4. Discussion

### 4.1. Carotid Intima-Media Thickness and Cardiovascular Risk

CIMT is a widely established surrogate marker of subclinical atherosclerosis and cardiovascular risk [12,13,14,15,16]. In the general population, elevated CIMT correlates with an increased incidence of coronary artery disease, myocardial infarction, AF, and stroke [20,32,33,34]. An increased CIMT indicates alterations in the arterial wall, including lipid accumulation, inflammation, smooth muscle proliferation, and endothelial dysfunction, all of which contribute to plaque formation, rupture, and thromboembolism [12,13,14,15,16,17,18,19]. It therefore reflects a patient`s cumulative burden of cardiovascular risk factors, essentially serving as a marker for the degree of systemic atherosclerosis [12,13,14,15,16,17,18,19]. Consequently, each incremental increase in CIMT correlates with a greater likelihood of adverse cardiovascular events [12,13,14,15,16,17,18,19]. The European Society of Cardiology guidelines define a threshold of CIMT > 0.9 mm as abnormal, signifying an increased risk of cardiovascular events [17,18,19].

As data regarding the clinical relevance of CIMT in HTX recipients are limited [23,24], we investigated the effects of pre-transplant CIMT on post-transplant outcomes in 311 HTX recipients. Pre-transplant CIMT > 0.9 mm was present in a small subgroup of 11.9% of our cohort, and these patients had significantly more atherosclerotic risk factors (older recipient age, arterial hypertension, dyslipidemia, diabetes mellitus, history of smoking, and COPD) than patients with a pre-transplant CIMT ≤ 0.9 mm, consistent with the notion that CIMT integrates multiple risk factor effects [12,13,14,15,16,17,18,19]. In addition, patients with a pre-transplant CIMT > 0.9 mm significantly more often received CABG surgery before HTX (32.2% versus 10.6%) and more frequently suffered from ischemic CMP as principal diagnosis for HTX (64.9% versus 30.6%). This further supports the role of CIMT as a surrogate marker of systemic atherosclerosis and cardiovascular risk [12,13,14,15,16,17,18,19].

The cardiovascular risk profile and the concomitant medications of HTX recipients with increased pre-transplant CIMT have notably not been well characterized prior to this study. We found no significant differences between patients with a pre-transplant CIMT ≤ 0.9 mm and patients with a pre-transplant CIMT > 0.9 mm regarding the administration of acetylsalicylic acid, antihypertensive agents, or statins, highlighting the already high cardiovascular risk profile in this specific patient cohort [25,26,27,28,29,30].

### 4.2. Mortality and Causes of Death After Heart Transplantation

Post-transplant survival continues to be limited by several factors, including graft failure, acute rejection, infection/sepsis, malignancy, and thromboembolic events/bleeding [35]. Cardiovascular diseases such as arterial hypertension, dyslipidemia, diabetes mellitus, and history of smoking are often more prevalent in HTX recipients than in the general population and can lead to reduced post-transplant survival by contributing to the pathogenesis of graft failure and thromboembolic events/bleeding [6,7,8,9,10,11,36].

Consistent with this, our findings show that a pre-transplant CIMT > 0.9 mm—a marker of systemic atherosclerosis and cardiovascular risk—identified HTX recipients with markedly worse short- and long-term survival after HTX. Patients with a pre-transplant CIMT > 0.9 mm had a significantly higher 30-day mortality after HTX (18.9% versus 3.6%, *p* < 0.001) and 10-year mortality after HTX (81.1% versus 41.2%, *p* < 0.001). Notably, the survival difference between patients with a pre-transplant CIMT > 0.9 mm and those ≤ 0.9 mm arose already within the first post-transplant year. Analysis of the causes of death in our cohort provided insights into this reduced survival after HTX. Patients with a pre-transplant CIMT > 0.9 mm had significantly higher rates of death due to graft failure, infection/sepsis, and thromboembolic events/bleeding. Notably, 24.3% of patients with a pre-transplant CIMT > 0.9 mm died from graft failure within ten years after HTX compared to only 10.6% of patients with a pre-transplant CIMT ≤ 0.9 mm (*p* = 0.017). We suspect that some of these graft failures may represent CAV-related graft failure as the same cardiovascular risk factors that drive CIMT (hypertension, dyslipidemia, diabetes, and smoking) are also known contributors to CAV development [7,8,36]. It is therefore plausible that HTX recipients with extensive atherosclerosis are predisposed to more rapid CAV progression, leading to graft failure and death [7,8,36].

Likewise, infection/sepsis accounted for a larger share of deaths in patients with a pre-transplant CIMT > 0.9 mm within ten years after HTX (37.8% versus 20.4%, *p* = 0.017). This may reflect the fact that patients with a pre-transplant CIMT > 0.9 mm in our study were older and more likely to have diabetes mellitus and COPD, factors associated with frailty, impaired immunity, and higher risk for infections [29,30].

We also observed more fatal thromboembolic events/bleeding in patients with a pre-transplant CIMT > 0.9 mm within ten years after HTX (10.8% versus 2.9%, *p* = 0.019), which aligns with a higher incidence of post-transplant AF and stroke in patients with increased CIMT [20,32,34].

In contrast, there was no significant difference in deaths from acute rejection between groups, suggesting that immune-mediated graft loss was not a differentiator of outcomes in this cohort. Furthermore, a pre-transplant CIMT > 0.9 mm remained an independent risk factor for a more than twofold increased mortality within ten years after HTX, even after adjusting for age and several cardiovascular risk factors. Importantly, even though ischemic cardiomyopathy etiology was more common in patients with a pre-transplant CIMT > 0.9 mm, in our multivariate analysis, pre-transplant CIMT > 0.9 mm retained significance for increased mortality, whereas ischemic cardiomyopathy etiology did not. This suggests that extensive pre-transplant atherosclerosis confers an elevated mortality risk that is not fully captured by conventional risk factors alone. In addition, we could confirm that COPD is an independent risk factor for reduced post-transplant survival, which has been suggested before [29].

Overall, our data indicate that excess mortality among patients with a pre-transplant CIMT > 0.9 mm is largely attributable to multi-factorial, non-rejection causes linked to a pro-atherosclerotic and frail phenotype. However, a pre-transplant CIMT > 0.9 mm should not be viewed in isolation or used as a sole criterion but rather considered alongside all other factors. This underscores the importance of optimizing recipient selection and aggressively managing comorbid conditions. From a clinical standpoint, identifying HTX candidates with a pre-transplant CIMT > 0.9 mm could prompt closer surveillance, prophylactic strategies (intensified infection vigilance), and optimized pharmacological therapy (hypertension, dyslipidemia, diabetes) to mitigate these risks.

### 4.3. Cardiovascular Events After Heart Transplantation

Early post-transplant AF and stroke are clinically significant complications and have been associated with increased morbidity and mortality in patients after HTX [26,37]. Published rates of early post-transplant AF range from 7.9% to 18.2% depending on the observed post-transplant interval [26,38,39]. Approximately 10% of HTX recipients experience a post-transplant stroke, with the majority of cases classified as ischemic in nature [37]. The etiology of post-transplant AF and stroke is multifactorial and includes factors such as age, surgical HTX technique, prolonged ischemic time, graft failure, COPD, and diabetes [26,37,38,39].

Previous studies in non-transplant patients have linked an increased CIMT to a higher occurrence of AF and stroke, suggesting that systemic vascular stiffness and atherosclerotic burden may play an important role in the pathogenesis of AF [20,32,34]. This is in line with our findings in HTX recipients as in our study, 27.0% of patients with a pre-transplant CIMT > 0.9 mm developed AF within 30 days of HTX versus 10.9% of patients with a pre-transplant CIMT ≤ 0.9 mm. This two-fold to three-fold increase suggests that the recipient’s high systemic atherosclerotic burden may create a pro-arrhythmic milieu provoking AF, even though the transplanted heart comes from a healthy donor (in a cardiovascular sense). In addition, HTX recipients with pre-existing AF before HTX are more likely to develop early post-transplant AF as an expression of a chronic disease state [26].

Arguably the most striking finding is the pronounced disparity in early post-transplant stroke incidence. Within 30 days after HTX, 10.8% of patients with a pre-transplant CIMT > 0.9 mm experienced a stroke, compared to only 1.1% of those with a pre-transplant CIMT ≤ 0.9 mm. Several factors could explain this disparity. Post-transplant AF is one plausible mediator as AF is a well-known cause of cardioembolic stroke [37], and the excess of AF in patients with a pre-transplant CIMT > 0.9 mm likely contributed to their higher stroke rate. Beyond AF, an elevated pre-transplant CIMT reflects systemic atherosclerosis, often encompassing unstable plaques that may become dislodged perioperatively. This is consistent with broad epidemiologic data linking increased CIMT to higher stroke risk in non-transplant populations [20].

In summary, the observed strong association between a pre-transplant CIMT > 0.9 mm and worse post-transplant outcomes indicates that careful assessment of CIMT during the HTX evaluation process could help identify high-risk HTX candidates who harbor an excessive atherosclerotic burden not captured by traditional risk factors alone.

## 5. Study Limitations

Our findings are based on data from a large, single-center registry (Heidelberg HTX Registry). As this study design inherently carries certain limitations, the results should be interpreted with caution and viewed within the context of the broader existing literature. The retrospective design introduces inherent limitations, particularly around potential confounding factors and selection bias. Despite multivariate adjustments, residual confounding cannot be fully excluded. Nevertheless, to our knowledge, this is the most comprehensive analysis to date examining the association between pre-transplant CIMT > 0.9 mm and outcomes following HTX. The analysis was conducted using detailed clinical data from 311 HTX recipients who underwent standardized treatment and follow-up protocols, thereby minimizing potential selection bias and confounding factors [25,26,27,28,29,30].

To achieve a robust sample size for reliable statistical analysis, we included patients who underwent HTX at the Heidelberg Heart Center between 2002 and 2014, with a follow-up duration of up to 10 years. Given the extended study period, temporal changes in surgical techniques and medical management (i.e., an era effect) could have influenced the outcomes. To address this, we conducted a sensitivity analysis restricted to patients treated with tacrolimus and mycophenolic acid, a standard immunosuppressive regimen adopted at Heidelberg Heart Center from 2006 onward. The consistency of results in this subgroup supports the robustness of our overall findings [25,26,27,28,29,30].

Ideally, stratification of patients would have happened by pre-transplant CIMT and presence of carotid plaques to further refine cardiovascular risk stratification. Given the retrospective nature of our study, we were unable to analyze carotid plaque burden or post-transplant CIMT progression over time, because detailed carotid plaque data were not available for all patients in our cohort. Importantly, our results should be considered hypothesis-generating, particularly in relation to post-transplant survival, which is influenced by multiple factors. While we observed an association between pre-transplant CIMT > 0.9 mm and increased post-transplant mortality, these findings do not establish causality. Further validation through large-scale, multicenter prospective studies is warranted to confirm these observations and better elucidate the underlying mechanisms [25,26,27,28,29,30].

## 6. Conclusions

CIMT is a well-established risk marker for adverse cardiovascular events in the general population. However, its prognostic significance in HTX recipients remains unclear. In this retrospective, single-center observational study, we examined the association between increased pre-transplant CIMT > 0.9 mm and post-transplant outcomes in a cohort of 311 patients who underwent HTX at the Heidelberg Heart Center between 2002 and 2014. Among these, 37 patients (11.9%) had a pre-transplant CIMT > 0.9 mm. Patients with a pre-transplant CIMT > 0.9 mm demonstrated a significantly higher 10-year post-transplant mortality compared to those with a CIMT ≤ 0.9 mm (81.1% versus 41.2%, *p* < 0.001). Additionally, this group showed increased rates of death due to graft failure (24.3% versus 10.6%, *p* = 0.017) and thromboembolic events/bleeding (10.8% vs. 2.9%, *p* = 0.019). Multivariate analysis identified a pre-transplant CIMT > 0.9 mm as an independent risk factor for 10-year mortality (HR: 2.599, 95% CI: 1.683–4.014, *p* < 0.001). Analysis of secondary outcomes revealed a significantly higher rate of 30-day post-transplant AF (27.0% versus 10.9%, *p* = 0.006) and 30-day stroke after HTX (10.8% versus 1.1%, *p* < 0.001) in patients with a pre-transplant CIMT > 0.9 mm.

In summary, our findings indicate that a pre-transplant CIMT > 0.9 mm is associated with increased early (AF and stroke) as well as long-term (mortality, graft failure, and thromboembolic events/bleeding) adverse outcomes after HTX. These results suggest that a pre-transplant CIMT > 0.9 mm may serve as a valuable and easily accessible tool for identifying HTX candidates at elevated cardiovascular risk. Such patients might benefit from intensified surveillance and risk factor management in the peri-transplant and post-transplant period. If our findings are confirmed in larger studies, a pre-transplant CIMT > 0.9 mm might warrant careful consideration in HTX candidate selection given the high early mortality risk—although at present, we recommend using a pre-transplant CIMT > 0.9 mm primarily to identify high-risk patients for closer monitoring and risk factor optimization, rather than as an exclusion criterion.

## Figures and Tables

**Table 1:** Demographic and clinical characteristics.

Parameter	All Patients (*n* = 311)	CIMT ≤ 0.9 mm (*n* = 274)	CIMT > 0.9 mm (*n* = 37)	Difference	95% CI	*p*-Value	
Recipient data							
Age (years), mean ± SD	51.9 ± 10.6	51.3 ± 10.9	56.5 ± 6.2	5.2	2.8–7.6	<0.001	*
Male sex, *n* (%)	239 (76.8%)	208 (75.9%)	31 (83.8%)	7.9%	−5.0–20.8%	0.287	
BMI (kg/m^2^), mean ± SD	25.1 ± 4.4	25.0 ± 4.4	25.4 ± 3.9	0.4	−1.0–1.8	0.558	
Arterial hypertension, *n* (%)	174 (55.9%)	146 (53.3%)	28 (75.7%)	22.4%	7.4–37.4%	0.010	*
Dyslipidemia, *n* (%)	198 (63.7%)	169 (61.7%)	29 (78.4%)	16.7%	2.2–31.2%	0.047	*
Diabetes mellitus, *n* (%)	103 (33.1%)	84 (30.7%)	19 (51.4%)	20.7%	3.7–37.7%	0.012	*
Peripheral artery disease, *n* (%)	28 (9.0%)	22 (8.0%)	6 (16.2%)	8.2%	−4.1–20.5%	0.102	
COPD, *n* (%)	82 (26.4%)	62 (22.6%)	20 (54.1%)	31.5%	14.7–48.3%	<0.001	*
History of smoking, *n* (%)	178 (57.2%)	150 (54.7%)	28 (75.7%)	21.0%	6.0–36.0%	0.016	*
Chronic kidney disease ^, *n* (%)	173 (55.6%)	149 (54.4%)	24 (64.9%)	10.5%	−6.0–27.0%	0.228	
eGFR (ml/min/1.73 m^2^), mean ± SD	58.3 ± 23.6	59.0 ± 24.3	53.3 ± 16.7	5.7	−0.4–11.8	0.073	
Previous open-heart surgery							
Overall open-heart surgery, *n* (%)	87 (28.0%)	69 (25.2%)	18 (48.6%)	23.4%	6.5–40.3%	0.003	*
CABG surgery, *n* (%)	41 (13.2%)	29 (10.6%)	12 (32.4%)	21.8%	6.3–37.3%	<0.001	*
Other surgery °, *n* (%)	34 (10.9%)	30 (10.9%)	4 (10.8%)	0.1%	−10.6–10.8%	0.980	
VAD surgery, *n* (%)	18 (5.8%)	16 (5.8%)	2 (5.4%)	0.4%	−7.4–8.2%	0.915	
Principal diagnosis for HTX							
Ischemic CMP, *n* (%)	108 (34.7%)	84 (30.6%)	24 (64.9%)	34.3%	18.0–50.6%	<0.001	*
Non-ischemic CMP, *n* (%)	147 (47.3%)	138 (50.4%)	9 (24.3%)	26.1%	11.1–41.1%	0.003	*
Valvular heart disease, *n* (%)	14 (4.5%)	12 (4.4%)	2 (5.4%)	1.0%	−6.7–8.7%	0.778	
Cardiac amyloidosis, *n* (%)	42 (13.5%)	40 (14.6%)	2 (5.4%)	9.2%	−0.8–19.2%	0.125	
Donor data							
Age (years), mean ± SD	45.0 ± 12.5	44.9 ± 12.6	46.2 ± 12.0	1.3	−2.8–5.4	0.528	
Male sex, *n* (%)	95 (30.5%)	82 (29.9%)	13 (35.1%)	5.2%	−11.1–21.5%	0.519	
BMI (kg/m^2^), mean ± SD	25.0 ± 4.7	25.1 ± 4.8	24.2 ± 3.4	0.9	−0.4–2.2	0.193	
Transplant sex mismatch							
Mismatch, *n* (%)	163 (52.4%)	143 (52.2%)	20 (54.1%)	1.9%	−15.2–19.0%	0.831	
Donor (m) to recipient (f), *n* (%)	9 (2.9%)	8 (2.9%)	1 (2.7%)	0.2%	−5.4–5.8%	0.941	
Donor (f) to recipient (m), *n* (%)	154 (49.5%)	135 (49.3%)	19 (51.4%)	2.1%	−15.1–19.3%	0.812	
Perioperative data							
High-urgent listing status, *n* (%)	247 (79.4%)	218 (79.6%)	29 (78.4%)	1.2%	−12.9–15.3%	0.867	
Ischemic time (min), mean ± SD	253.0 ± 58.4	253.6 ± 58.4	248.0 ± 58.9	5.6	−14.6–25.8	0.586	
Biatrial anastomosis, *n* (%)	4 (1.3%)	4 (1.5%)	0 (0.0%)	1.5%	−0.1–3.1%	0.459	
Bicaval anastomosis, *n* (%)	116 (37.3%)	101 (36.8%)	15 (40.5%)	3.7%	−13.1–20.5%	0.664	
Total orthotopic anastomosis, *n* (%)	191 (61.4%)	169 (61.7%)	22 (59.5%)	2.2%	−14.6–19.0%	0.795	

Abbreviations: BMI = body mass index; CABG = coronary artery bypass graft; CI = confidence interval; CIMT = carotid intima-media thickness; CMP = cardiomyopathy; COPD = chronic obstructive pulmonary disease; f = female; eGFR = estimated glomerular filtration rate; HTX = heart transplantation; m = male; *n* = number; SD = standard deviation; VAD = ventricular assist device; ^ = eGFR < 60 mL/min/1.73 m^2^; ° = congenital, valvular, or ventricular surgery; * = statistically significant (*p* < 0.050).

**Table 2:** Initial post-transplant medications.

Parameter	All Patients (*n* = 311)	CIMT ≤ 0.9 mm (*n* = 274)	CIMT > 0.9 mm (*n* = 37)	Difference	95% CI	*p*-Value
Immunosuppressive drug therapy						
Cyclosporine A, *n* (%)	86 (27.7%)	72 (26.3%)	14 (37.8%)	11.5%	−5.0–28.0%	0.140
Tacrolimus, *n* (%)	225 (72.3%)	202 (73.7%)	23 (62.2%)	11.5%	−5.0–28.0%	0.140
Azathioprine, *n* (%)	10 (3.2%)	9 (3.3%)	1 (2.7%)	0.6%	−5.0–6.2%	0.851
Mycophenolic acid, *n* (%)	301 (96.8%)	265 (96.7%)	36 (97.3%)	0.6%	−5.0–6.2%	0.851
Steroids, *n* (%)	311 (100.0%)	274 (100.0%)	37 (100.0%)	0.0%	n.a.	n.a.
Concomitant medications						
ASA, *n* (%)	42 (13.5%)	39 (14.2%)	3 (8.1%)	6.1%	−3.6–15.8%	0.306
Beta blocker, *n* (%)	71 (22.8%)	66 (24.1%)	5 (13.5%)	10.6%	−1.5–22.7%	0.150
Ivabradine, *n* (%)	39 (12.5%)	36 (13.1%)	3 (8.1%)	5.0%	−4.7–14.7%	0.386
Calcium channel blocker, *n* (%)	85 (27.3%)	71 (25.9%)	14 (37.8%)	11.9%	−4.6–28.4%	0.127
ACE inhibitor/ARB, *n* (%)	132 (42.4%)	114 (41.6%)	18 (48.6%)	7.0%	−10.1–24.1%	0.416
Diuretic, *n* (%)	311 (100.0%)	274 (100.0%)	37 (100.0%)	0.0%	n.a.	n.a.
Statin, *n* (%)	194 (62.4%)	172 (62.8%)	22 (59.5%)	3.3%	−13.5–20.1%	0.696
Gastric protection drug ^†^, *n* (%)	311 (100.0%)	274 (100.0%)	37 (100.0%)	0.0%	n.a.	n.a.

Abbreviations: ACE inhibitor = angiotensin-converting-enzyme inhibitor; ARB = angiotensin II receptor blocker; ASA = acetylsalicylic acid; CI = confidence interval; CIMT = carotid intima-media thickness; *n* = number; n.a. = not applicable; ^†^ = gastric protection drug defined as proton-pump inhibitor (PPI) or histamine receptor (H_2_) blocker.

**Table 3:** Post-transplant primary outcome.

Parameter	All Patients (*n* = 311)	CIMT ≤ 0.9 mm (*n* = 274)	CIMT > 0.9 mm (*n* = 37)	Difference	95% CI	*p*-Value	
30-day mortality after HTX, *n* (%)	17 (5.5%)	10 (3.6%)	7 (18.9%)	15.3%	2.5–28.1%	<0.001	*
1-year mortality after HTX, *n* (%)	70 (22.5%)	48 (17.5%)	22 (59.5%)	42.0%	25.6–58.4%	<0.001	*
2-year mortality after HTX, *n* (%)	82 (26.4%)	56 (20.4%)	26 (70.3%)	49.9%	34.4–65.4%	<0.001	*
5-year mortality after HTX, *n* (%)	106 (34.1%)	79 (28.8%)	27 (72.9%)	44.1%	28.8–59.4%	<0.001	*
10-year mortality after HTX, *n* (%)	143 (46.0%)	113 (41.2%)	30 (81.1%)	39.9%	26.0–53.8%	<0.001	*

Abbreviations: CI = confidence interval; CIMT = carotid intima-media thickness; HTX = heart transplantation; *n* = number; * = statistically significant (*p* < 0.050).

**Table 4:** Causes of death after HTX.

(**a**) Within 5 years after HTX.							
**Parameter**	**All Patients** **(*n* = 311)**	**CIMT ≤ 0.9 mm** **(*n* = 274)**	**CIMT > 0.9 mm** **(*n* = 37)**	**Difference**	**95% CI**	***p*-Value**	
Graft failure, *n* (%)	30 (9.6%)	22 (8.0%)	8 (21.6%)	13.6%	0.4–26.8%	0.009	*
Acute rejection, *n* (%)	2 (0.6%)	2 (0.7%)	0 (0.0%)	0.7%	−0.3–1.7%	0.602	
Infection/Sepsis, *n* (%)	59 (19.0%)	45 (16.4%)	14 (37.8%)	21.4%	5.2–37.6%	0.002	*
Malignancy, *n* (%)	7 (2.3%)	6 (2.2%)	1 (2.7%)	0.5%	−5.0–6.0%	0.843	
Thromboembolic event/bleeding, *n* (%)	8 (2.6%)	4 (1.5%)	4 (10.8%)	9.3%	0.8–17.8%	0.001	*
All causes, *n* (%)	106 (34.1%)	79 (28.8%)	27 (72.9%)	44.1%	28.8–59.4%	<0.001	*
(**b**) Within 10 years after HTX.							
**Parameter**	**All Patients** **(*n* = 311)**	**CIMT ≤ 0.9 mm** **(*n* = 274)**	**CIMT > 0.9 mm** **(*n* = 37)**	**Difference**	**95% CI**	***p*-Value**	
Graft failure, *n* (%)	38 (12.2%)	29 (10.6%)	9 (24.3%)	13.7%	0.6–26.8%	0.017	*
Acute rejection, *n* (%)	3 (1.0%)	2 (0.7%)	1 (2.7%)	2.0%	−3.3–7.3%	0.249	
Infection/Sepsis, *n* (%)	70 (22.5%)	56 (20.4%)	14 (37.8%)	17.4%	1.1–33.7%	0.017	*
Malignancy, *n* (%)	20 (6.4%)	18 (6.6%)	2 (5.4%)	1.2%	−6.7–9.1%	0.786	
Thromboembolic event/bleeding, *n* (%)	12 (3.9%)	8 (2.9%)	4 (10.8%)	7.9%	0.2–15.6%	0.019	*
All causes, *n* (%)	143 (46.0%)	113 (41.2%)	30 (81.0%)	39.8%	25.9–53.7%	<0.001	*

Abbreviations: CI = confidence interval; CIMT = carotid intima-media thickness; HTX = heart transplantation; *n* = number; * = statistically significant (*p* < 0.050).

**Table 5:** Multivariate analysis for mortality after HTX.

(**a**) Within 5 years after HTX.				
**Parameter**	**Hazard Ratio**	**95% CI**	***p*-Value**	
Recipient age (years)	1.022	0.998–1.046	0.068	
Arterial hypertension (in total)	1.235	0.660–2.310	0.509	
Dyslipidemia (in total)	0.632	0.338–1.179	0.149	
Diabetes mellitus (in total)	1.435	0.949–2.171	0.087	
COPD (in total)	4.748	2.850–7.910	<0.001	*
History of smoking (in total)	0.588	0.336–1.028	0.062	
CABG surgery ^ (in total)	0.993	0.532–1.852	0.981	
Ischemic CMP ° (in total)	0.970	0.535–1.760	0.921	
CIMT > 0.9 mm (in total)	2.899	1.802–4.664	<0.001	*
(**b**) Within 10 years after HTX.				
**Parameter**	**Hazard Ratio**	**95% CI**	***p*-Value**	
Recipient age (years)	1.013	0.994–1.033	0.192	
Arterial hypertension (in total)	1.163	0.674–2.008	0.588	
Dyslipidemia (in total)	0.667	0.389–1.143	0.141	
Diabetes mellitus (in total)	1.223	0.852–1.756	0.275	
COPD (in total)	4.695	3.098–7.115	<0.001	*
History of smoking (in total)	0.843	0.530–1.341	0.471	
CABG surgery ^ (in total)	1.212	0.713–2.059	0.478	
Ischemic CMP ° (in total)	0.943	0.561–1.584	0.823	
CIMT > 0.9 mm (in total)	2.599	1.683–4.014	<0.001	*

Abbreviations: CABG = coronary artery bypass graft; CI = confidence interval; CIMT = carotid intima-media thickness; CMP = cardiomyopathy; COPD = chronic obstructive pulmonary disease; HTX = heart transplantation; ^ = before HTX; ° = as principal diagnosis for HTX; * = statistically significant (*p* < 0.050).

**Table 6:** Post-transplant secondary outcomes.

Parameter	All Patients (*n* = 311)	CIMT ≤ 0.9 mm (*n* = 274)	CIMT > 0.9 mm (*n* = 37)	Difference	95% CI	*p*-Value	
30-day atrial fibrillation after HTX, *n* (%)	40 (12.9%)	30 (10.9%)	10 (27.0%)	16.1%	1.3–30.9%	0.006	*
30-day rejection episode after HTX, *n* (%)	40 (12.9%)	36 (13.1%)	4 (10.8%)	2.3%	−8.5–13.1%	0.691	
30-day TIA after HTX, *n* (%)	0 (0.0%)	0 (0.0%)	0 (0.0%)	0.0%	n.a.	n.a.	
30-day stroke after HTX, *n* (%)	7 (2.3%)	3 (1.1%)	4 (10.8%)	9.7%	0.4–19.0%	<0.001	*

Abbreviations: CI = confidence interval; CIMT = carotid intima-media thickness; HTX = heart transplantation; *n* = number; n.a. = not applicable; TIA = transient ischemic attack; * = statistically significant (*p* < 0.050).

## Data Availability

The original contributions presented in this study are included in the article; further inquiries can be directed to the corresponding author.
